# Pancake kidney with cysts and a single ureter

**DOI:** 10.1590/0100-3984.2015.0063

**Published:** 2016

**Authors:** Renata Mendes da Silva, Moaci Ferreira de Morais Júnior, Francisco Edward Mont'Alverne Filho

**Affiliations:** 1 Universitary Hospital, Universidade Federal do Piauí (UFPI), Teresina, PI, Brazil.

*Dear Editor*,

We report the case of a 56-year-old male patient who had undergone cholecystectomy with biliary-enteric anastomosis for the treatment of choledocholithiasis and was referred by a general surgeon for computed tomography (CT) at the University Hospital - Universidade Federal do Piauí, Brazil, because of the intraoperative finding of a pulsatile mass in the abdomen. The patient had no other comorbidities and was taking no medications at the time of referral.

**Figure 1:**
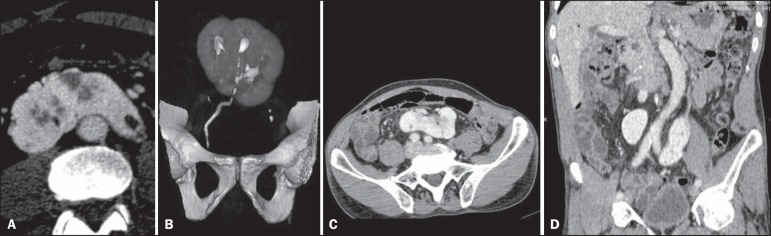
**A:** Axial CT image of the abdomen in the arterial phase showing pancake kidney with cysts. **B:** Three-dimensional CT reconstruction of the abdomen (excretory phase) showing a single ureter. **C,D:** Axial CT images and coronal CT reconstruction of the abdomen (portal phase), showing a single, flat, medial, non-reniform mass, at the level of the aortic bifurcation.

A contrast-enhanced abdominal CT scan identified a pancake kidney with cysts (Figure 1A), and three-dimensional CT reconstruction with contrast (excretory phase) revealed that there was a single ureter (Figure 1B). The axial CT images and coronal CT reconstruction (portal phase) images of the abdomen showed a single, flat, medial, non-reniform mass in the region of the aortoiliac bifurcation, characteristic of the anomaly known as pancake kidney (Figures 1C and 1D).

Pancake kidney is a rare congenital urinary tract anomaly, the exact incidence of which is unknown^(1)^. Like other abnormalities involving renal fusion, pancake kidney is most commonly found in males, at a ratio of 2-3:1, and can be diagnosed at any age.^(2)^

The pancake kidney malformation results from complete medial fusion of the metanephric blastema at an early stage of embryonic development and is characterized by a single, flat, nonreniform mass, in a medial position within the pelvic cavity or at the level of the aortic bifurcation. The renal collecting system is anterior and typically drains via two ureters or, less commonly, via a single ureter. The renal vasculature is also anomalous; blood flow can be supplied by multiple branches of the internal and external iliac arteries or of the abdominal aorta^(3)^.

In most cases, pancake kidney is asymptomatic but can be accompanied by nephrolithiasis, hydronephrosis, and vesicoureteral reflux resulting in recurrent urinary infections, all of which are attributable to the anomalous rotation of the collecting system and the short ureters, which are prone to stasis and obstruction, as well as by renovascular hypertension, ureteropelvic junction stenosis, anomalous implantation of the renal pelvis, and polycystic kidney disease^(1,4)^. Among individuals with pancake kidney, the incidence of neoplasms, Wilms tumor in particular, is higher^(5)^.

A little more than 20 cases of pancake kidney have been described in the literature, and a single ureter was reported in fewer than 10 of those cases^(6,7)^. Early identification of renal abnormalities is important to the investigation of associated conditions and for the differential diagnosis of pelvic masses, in order to preventing unnecessary injury or removal^(3,6)^. Here, we have reported another case of the rare anomaly pancake kidney, accompanied by cysts and with a single ureter, in a patient who was asymptomatic and was diagnosed after an incidental intraoperative finding.
